# *Aeromonas punctata* derived depolymerase improves susceptibility of *Klebsiella pneumoniae* biofilm to gentamicin

**DOI:** 10.1186/s12866-015-0455-z

**Published:** 2015-06-11

**Authors:** Shruti Bansal, Kusum Harjai, Sanjay Chhibber

**Affiliations:** Department of Microbiology, Panjab University, Sector-14, Chandigarh, 160014 India

**Keywords:** Anti-biofilm enzyme, Bacterial depolymerase, Phage depolymerase, Enzyme kinetics, Gentamicin

## Abstract

**Background:**

To overcome antibiotic resistance in biofilms, enzymes aimed at biofilm dispersal are under investigation. In the present study, applicability of an *Aeromonas punctata* derived depolymerase capable of degrading the capsular polysaccharide (CPS) of *Klebsiella pneumoniae,* in disrupting its biofilm and increasing gentamicin efficacy against biofilm was investigated.

**Results:**

Intact biofilm of *K. pneumoniae* was recalcitrant to gentamicin due to lack of antibiotic penetration. On the other hand, gentamicin could not act on disrupted biofilm cells due to their presence in clusters. However, when depolymerase (20 units/ml) was used in combination with gentamicin (10 μg/ml), dispersal of CPS matrix by enzyme facilitated gentamicin penetration across biofilm. This resulted in significant reduction (*p* < 0.05) in bacterial count in intact and disrupted biofilms. Reduction in CPS after treatment with depolymerase was confirmed by confocal microscopy and enzyme linked lectinosorbent assay. Furthermore, to substantiate our study, the efficacy of bacterial depolymerase was compared with a phage borne depolymerase possessing similar application against *K. pneumoniae*. Although both were used at same concentration i.e. 20 units/ml, but a higher efficacy of bacterial depolymerase particularly against older biofilms was visibly clear over its phage counterpart. This could be explained due to high substrate affinity (indicated by K_m_ value) and high turnover number (indicated by K_cat_ value) of the bacterial depolymerase (K_m_ = 89.88 μM, K_cat_ = 285 s^−1^) over the phage derived one (K_m_ = 150 μM, K_cat_ = 107 s^−1^).

**Conclusion:**

Overall the study indicated that, the *A. punctata* derived depolymerase possesses antibiofilm potential and improves gentamicin efficacy against *K. pneumoniae*. Moreover, it can serve as a potential substitute to phage borne depolymerases for treating biofilms formed by *K. pneumoniae*.

**Electronic supplementary material:**

The online version of this article (doi:10.1186/s12866-015-0455-z) contains supplementary material, which is available to authorized users.

## Background

Biofilm is a community of microorganisms embedded in a self produced polymeric matrix comprising of polysaccharides, proteins, glycopeptides, nucleic acids and lipids [1]. Currently, it is estimated that over 60 % of bacterial infections and up to 80 % of chronic infections involve microbial growth in biofilms [2]. *K. pneumoniae* is frequently encountered in hospital-acquired urinary tract infections (UTI), respiratory illnesses, burn wound infections, surgical site and catheter-related infections [3]. Mortality rates due to infections by this organism range between 25-60 % [4, 5]. Biofilm formation is a major virulence trait used by *K. pneumoniae* to colonize the human host [6]. The acidic CPS encasing the bacterium provides resistance against dessication, phagocytosis, killing by serum, induces cytotoxicity during infection of lung epithelial cells and helps in biofilm formation at the infection site [7, 8]. Recently, hypermucoviscous variants of *K. pneumoniae* with increased virulence have emerged in the Asia Pacific region and they are spreading across the globe [9]. This dense, sticky mix of negatively charged polysaccharides forms a three dimensional scaffold which can range in thickness upto 50 μm [10, 11]. It retards or blocks the penetration of antibiotics particularly positively charged aminoglycosides, thus restricting their access to the deeper layers [12]. This leads to sub-optimal levels of antibiotics reaching bacterial cells embedded in a biofilm. Exposure of bacteria to sub-MIC concentrations can further complicate the situation by enhancing CPS production and increasing the emergence of resistant strains. This has been observed in encapsulated isolates of *K. pneumoniae, Escherichia coli, P. aeruginosa* [13]. Multicellular nature of the polysaccharide also leads to creation of nutrient starved zones in the biofilm interior as a result of which some cells enter into a metabolically inactive state leading to inability of antibiotics to act on them [14]. Chemical disinfectants like nitric oxide, metal chelators like sodium citrate or tetrasodium-EDTA have been used to disrupt biofilms in healthcare and industrial settings but they leave toxic residues which have adverse effects [15]. To overcome drug resistance in biofilms, efforts are being directed towards developing anti-biofilm enzymes. Polysaccharide-degrading enzymes like α-amylases, dispersin B (DspB), alginate lyases, deoxyribonucleases (DNAses), phage depolymerases have been successfully used for preventing biofilm formation or disrupting the biofilms of *S. aureus, S. epidermidis, E. coli* and *P. aeruginosa, K. pneumoniae* [16]. Such enzymes offer several advantages: firstly, they are safe and have no side effects. Secondly, they do not require actively growing cells for their action and can act with the same frequency on metabolically active as well as inert cells [17, 18]. Thirdly, they are not directly bactericidal so they do not allow generation of resistant mutants. Although, the potential of phage depolymerases for disrupting the biofilms of *K. pneumoniae* and mediating the entry of antimicrobials has been recognized but*,* no report is available describing the use of enzymes from unrelated bacterial genera for disrupting *K. pneumoniae* polysaccharide in biofilms. The present study was undertaken to study the potential of a *Aeromonas punctata* derived depolymerase directed against K2 CPS of *K. pneumoniae* in disrupting the biofilms formed by this pathogen and improving its susceptibility to antibiotic. The biofilm disruption efficacy of *A. punctata* derived depolymerase with a previously characterized phage borne depolymerase against *K. pneumoniae* B5055 was also compared.

## Methods

### Bacterial strain, antibiotic

*K. pneumoniae* B5055 (O1:K2) [MTCC 5832] obtained from Dr. M. Trautman, Department of Medical Microbiology and Hygiene, University of Ulm, Germany was used in the present study. Maintenance of bacteria on nutrient agar slants and preparation of gentamicin (Himedia, India) was done as described previously [19]. Gentamicin was used at a final concentration of 10 μg/ml throughout the study.

### Enzyme

*Aeromonas punctata* (Accession no: KF158411) was cultivated in a statistically optimized media as standardized in our laboratory [20]. Cell free supernatant containing bacterial depolymerase directed against K2 CPS of *K. pneumoniae* B5055 was obtained. The enzyme was purified by anion exchange (DEAE) followed by gel filtration chromatography (Sephadex G100) [20]. CPS extracted from *K. pneumoniae* B5055 following the method of Hanlon et al. [21] was used as substrate for determining depolymerase activity by the method of Miller [22]. One unit of enzyme activity was defined as the micromoles of reducing sugars released ml^−1^ min^−1^ when the reaction was carried out at 37 °C and pH 7 for 60 min. Temperature and pH optima and stability for bacterial depolymerase are depicted in Additional file 1: Figure S1. Enzyme kinetics was determined for the purified protein using various substrate concentrations and K_m_, V_max_, k_cat_, k_cat_/K_m_ were calculated for the enzyme (Additional file 1: Figure S2).

For all experiments involving the use of *A. punctata* derived bacterial depolymerase for various applications, a previously characterized phage depolymerase capable of acting on K2 CPS of *K. pneumoniae* B5055 was also used. Phage depolymerase was extracted from a previously characterized, *K. pneumoniae* specific phage ‘KPO1K2’ (Verma et al. [23]) following the acid denaturation method of Reiger et al. [24]. Its purification and quantification was carried out in a similar manner as done for bacterial depolymerase. Both the enzymes were used at a concentration of 20 depolymerase units. All experiments involving the use of both the biological moieties were performed at 37 °C and pH 7 which was the temperature and pH optima for both the enzymes. The characteristics of phage depolymerase are depicted in Additional file 1: Figure S3 and Table S1.

### Capsule staining

Overnight culture of *K. pneumoniae* B5055 grown in nutrient broth at 37 °C was taken, cells were washed with PBS and diluted to obtain a count of 10^6^ CFU/ml. Bacterial depolymerase was added to a final concentration of 20 units/ml and cells were incubated for 30 min at 37 °C. Cells suspended in PBS acted as control. Both the bacterial suspensions were taken on a clean glass slides, mixed with a drop of safranin and a smear was made. It was then counterstained with crystal violet for 1 min, washed and examined under a light microscope.

### Penetration of gentamicin through intact biofilm

Penetration of gentamicin through biofilm formed by *K. pneumoniae* was determined according to the method of Anderl et al. [25] with modifications. Overnight culture of *K. pneumoniae* grown in nutrient broth was taken, cells were washed and diluted to obtain a bacterial count of 10^8^ CFU/ml. 10 μl of this bacterial culture was used to seed 25 mm polycarbonate discs (0.4 μm, Millipore) placed on nutrient agar plates. The drop was allowed to dry and plates were incubated at 37 °C. On days 1, 3, 5, 7, membrane biofilms were transferred to fresh nutrient agar plates containing a fresh lawn of *E. coli* ATCC 25922 (standard strain of *E. coli* used for quality control). On top of biofilm formed on 25 mm polycarbonate membrane, sterile 13 mm membrane (0.4 μm, Millipore) was carefully placed, such that the underlying biofilm was not disturbed. Sterile gentamicin disc (10 μg/ml, Hi-media) was then gently kept on the top of it and plates were incubated at 37 °C. Next day zone of clearance was measured on nutrient agar plate containing a lawn of *E. coli* ATCC 25922 [25]. Control assembly in which gentamicin disc was kept over sterile polycarbonate disc devoid of biofilm was also put alongwith the test. The zone of growth inhibition in control assembly was taken as 100 % penetration and used for determining the percentage penetration/retardation of antibiotic through biofilm. Experiment was repeated thrice in triplicates.

### Antibiotic susceptibility of planktonic, intact and disrupted biofilm cells

Intact biofilm was established in 96 well microtiter plate following the method described by Bedi et al. [26]. Briefly, 100 μl of nutrient broth and 100 μl of bacterial culture equivalent to 10^8^ CFU/ml (OD_600_ = 0.3) of *K. pneumoniae* was added to the wells and incubated overnight at 37 °C. After 24 h, supernatant was aspirated from the wells (*n* = 6) and 3 washings were given with sterile 0.85 % NaCl. Thereafter, gentamicin (10 μg/ml) was added to wells (*n* = 6). Biofilm in wells (*n* = 3) was disrupted using sterile toothpick according to the method described by El-Azizi et al. [27]. After an incubation of 6 h, wells with disrupted biofilm were aspirated (*n* = 3) while those with intact biofilm (*n* = 3) were scraped and added to sterile eppendorfs. The bacteria were vortexed and appropriately diluted in sterile 0.85 % NaCl. Bacterial count was estimated by plating the samples on nutrient agar plates. Percentage viability was calculated as ratio of cells in treated wells to cells in untreated, control wells multiplied by 100. In addition, to determination of viable count, the intact and disrupted biofilm were viewed under a fluorescent microscope (B2A filter set, Nikon, 40 X) after staining with Live/Dead staining Kit (Invitrogen) for 15 min. [Live/Dead staining kit contains 3.34 mM Syto 9 (stains live cells green) and 20 mM Propidium iodide (stains dead cells red)].

Simultaneously, antibiotic susceptibility of planktonic cells was determined by growing bacteria in 1 ml nutrient broth. After an incubation of 24 h, bacterial suspension was centrifuged, washed twice with 0.85 % NaCl and suspended in 1 ml fresh nutrient broth with gentamicin (10 μg/ml). After 6 h of incubation, bacteria were stained and percentage viability was determined by viable count.

### Treatment of intact and disrupted biofilm with depolymerase and antibiotic alone as well as in combination

Biofilm was formed in microtiter titer plate as described in section Antibiotic susceptibility of planktonic, intact and disrupted biofilm cells. On each day, supernatant was aspirated from all the wells and replaced with fresh nutrient broth. Three washings were given with sterile 0.85 % NaCl to a certain number of wells (*n* = 30). They were treated with various agents [gentamicin (10 μg/ml)/phage depolymerase (20 units/ml)/bacterial depolymerase (20 units/ml)/gentamicin + phage depolymerase/gentamicin + bacterial depolymerase, *n* = 6 each]. Biofilm in certain number of wells (*n* = 15) was disrupted using sterile toothpick by the method of El-Azizi et al. [27]. After an incubation of 6 h, wells with disrupted (*n* = 15) and intact biofilm (*n* = 15) were processed as described in section Antibiotic susceptibility of planktonic, intact and disrupted biofilm cells and viable count determined by plating the samples on nutrient agar plates. Biofilm was allowed to grow in different wells till 7 days. On each day, a set of 3 wells was processed for control, untreated intact and disrupted biofilm and the experiment was repeated thrice. The data was log transformed and for each time point mean of all replicate values was taken.

### Confocal laser-scanning microscopy (CLSM) of biofilm

Sterile plastic cover slips (18 x 18 mm) placed in 12 well mutidishes (Nunc) were inoculated with 1.5 ml culture of *K. pneumoniae* (1 × 10^8^ CFU/ml) and 1.5 ml nutrient broth and incubated at 37 °C. Each day, spent medium was removed and replaced with fresh nutrient broth. On 3^rd^ and 7^th^ day, the cover slips were washed thrice with 0.85 % NaCl, followed by treatment with 20 units/ml of either phage or bacterial depolymerase for 1 h at 37 °C. Thereafter, the cover slips with untreated and treated biofilms were stained with 300 mg/ml calcofluor white (Sigma) [calcofluor white binds to the sugar residues in the biofilm polysaccharide and stains them blue] and 0.1 % Syto 62 (Molecular Probes) [Syto 62, a nucleic acid staining dye stains the bacterial cells red] for 15 min each. These were then examined under a Inverted Olympus Fluoview CLSM (FV 1000, Olympus America Inc. NY, USA). All images were obtained with a 20X lens. Multiple images were collected for each set of experimental conditions; a representative image is presented for each group in the results section. Image analysis was done using z-series image stacks from four randomly chosen spots of each biofilm. The ratio of cell biomass to polysaccharide was calculated by dividing the integrated red fluorescence density with integrated blue fluorescence density using ImageJ version 1.46r [57].

### Quantitative estimation of CPS by enzyme linked lectinosorbent assay (ELLA)

CPS was quantified in the untreated/treated biofilm following the method of Strathmann et al. [28]. Briefly, intact biofilm was grown in microtiter plate and appropriately treated with phage/bacterial depolymerase as described in section Treatment of intact and disrupted biofilm with depolymerase and antibiotic alone as well as in combination. The biofilm was subsequently washed with phosphate buffer saline (PBS, 0.1 M, pH 7.2) twice and fixed with 200 μl of 95 % ethanol for 15 min. 100 μl of blocking solution [1 % w/v BSA in PBST (PBS plus 0.05 % Tween 20)] was added to the wells and incubated for 1 h at 37 °C. It was then pipetted out and 100 μl ConA–HRP (10 mg/ml) in 1 % BSA in PBST was added to wells. The microtiter plate was incubated for 30 min at 37 °C. Wells were washed thrice for 5 min with PBST. 100 μl of TMB substrate (BD Biosciences) was added and microtiter plate was incubated for 30 min at 37 °C. Reaction was stopped by adding 50 μl of 2 N H_2_SO_4_. CPS content (μg/ml) was measured in terms of peroxidase activity by taking absorbance at 450 nm. Experiment was performed in triplicate and repeated thrice.

### Statistical analysis

The effect of different treatments on biofilm was evaluated by applying one-way ANOVA and *P* < 0.05 was considered significant. Data was analyzed using SPSS 16.0.

## Results

The present study was conducted to evaluate the potential of *A. punctata* derived depolymerase to decapsulate *K. pneumoniae* B5055 and improve gentamicin efficacy against its biofilms.

### Enzyme kinetics for bacterial depolymerase

Bacterial depolymerase exhibited simple hyperbolic Michaelis-Menten kinetics. Lineweaver Burk plot was constructed and values depicting its kinetics i.e. K_m_, V_max_, k_cat_ and k_cat_/K_m_ were calculated as 89.88 μM, 43.35 μmol min^−1^, 285 s^−1^ and 3.17 s^−1^.μM^−1^ respectively (Additional file 1: Figure S2). Enzyme kinetics was also determined for the phage depolymerase and the values for K_m_, V_max_, k_cat_ and k_cat_/K_m_ are 150 μM, 20 μmol min^−1^, 107 s^−1^ and 0.71 μM^−1^ respectively. (Additional file 1: Table S1).

### Microscopic examination of bacteria after capsule staining

**Fig. 1:**
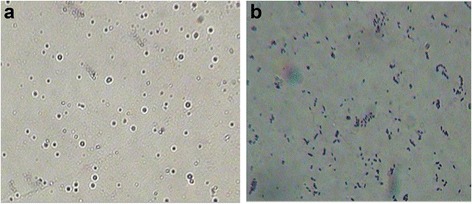
Microscopic appearance of *K. pneumoniae* B5055: (**a**) encapsulated untreated bacteria (**b**) depolymerase treated bacteria

A clear hollow depicting capsule was visible surrounding the untreated bacteria (Fig. 1a). On the other hand, depolymerase treated bacteria were devoid of any capsule (Fig. 1b).

### Penetration of gentamicin

Percentage retardation of gentamicin through colony biofilm of *K. pneumoniae* established on 25 mm polycarbonate disc was studied. As shown in Table 1, in comparison to inhibition zone observed on plates having membrane with no biofilm, a significant decrease in the diameter of inhibition zone (*p* < 0.05) was observed on plates in which membrane with colony biofilm was placed. Percentage antibiotic retardation increased with an increase in biofilm age. Retardation of gentamicin was 26.66 % for 1 day old biofilm. It progressively increased to 86.66 % for 7 day old biofilm (Table 1).

**Table 1:** Penetration of gentamicin (10 μg/ml) through biofilm of *K. pneumoniae* B5055

Biofilm age (Days)	Inhibition zones (mm) observed following penetration of gentamicin through disc placed on		% Retardation
	Membrane with no biofilm	Membrane with colony biofilm	
1	15 ± 0.06	11 ± 0.14	26.66 ± 0.17
3	15 ± 0.08	7 ± 0.18	53.33 ± 0.18
5	15 ± 0.12	3 ± 0.12	80.06 ± 0.15
7	15 ± 0.05	2 ± 0.13	86.66 ± 0.20

The zone of growth inhibition observed on plates with discs having no biofilms was taken as 100 % penetration and used to determine the percentage retardation of gentamicin through biofilms. Data is presented as mean ± SD. The experiment was performed thrice in triplicates

### Antibiotic susceptibility of planktonic, intact and disrupted biofilm cells

**Fig. 2:**
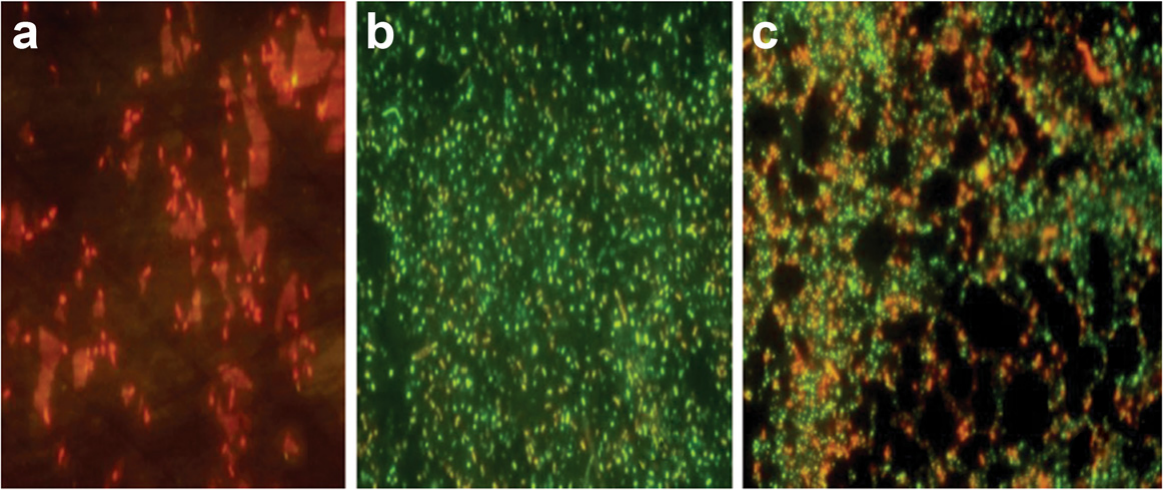
Planktonic cells (**a**) Intact biofilm (**b**) and Disrupted biofilm (**c**) incubated for 6 h with gentamicin (10 μg/ml), stained with LIVE/DEAD BacLight bacterial viability kit and visualized under a fluorescent microscope. Magnification: 40X, Scale:20 μm

Percentage viability of planktonic cells, intact and disrupted biofilm cells treated with gentamicin was 10 %, 100 % and 99.6 % respectively. Visualization of gentamicin treated cells under a fluorescent microscope indicated resistance of intact biofilm as a great number of green coloured viable cells (Syto 9 stained; Fig. 2b) were observed. The disrupted biofilm also consisted of large clumps of mostly viable, green cells (Fig. 2c). In contrast, all planktonic cells were dead as they acquired red color (PI stained, Fig. 2a). This indicated that, the disrupted biofilm cells were more or less similar in their antibiotic susceptibility pattern to bacteria in a intact biofilm.

### Effect of depolymerase and antibiotic alone as well as in combination on intact biofilm

**Fig. 3:**
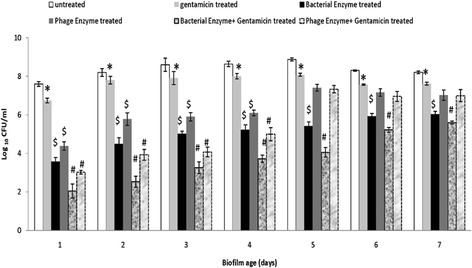
Bacterial count (Log_10_CFU/ml) following treatment of intact biofilm of *K. pneumoniae* grown in microtiter plate, with gentamicin (10 μg/ml)/phage/bacterial enzyme (20 units/ml) alone as well as in combination. The experiment was performed thrice in triplicate. Error bars = ± SD; *n* = 3 [*p > 0.05 (gentamicin treated vs untreated), ^$^ *p* < 0.05 (bacterial/phage enzyme treated vs untreated), ^#^ *p* < 0.05 (bacterial enzyme + gentamicin/phage enzyme + gentamicin treated vs untreated)]. Denatured bacterial/phage depolymerase heated at 75 °C for 10 min were used as controls for biofilm treatment. But no effect on CPS content was observed

The efficacy of depolymerase on gentamicin penetration and bacterial count was studied in intact biofilm. No significant difference (*p* > 0.05) was observed in average bacterial count of gentamicin treated biofilm and untreated biofilm (Fig. 3). This indicated the inability of antibiotic alone to tackle biofilm. When bacterial depolymerase was used alone, an average reduction of 3.261 ± 0.14 log in bacterial count was observed in comparison to untreated biofilm (Fig. 3). In contrast, an average reduction of 2.242 ± 0.21 log was observed after treatment with phage depolymerase alone (Fig. 3). Treatment with gentamicin and phage or bacterial depolymerase resulted in significant reduction (*p* < 0.05) of 4.259 ± 0.34 log and 5.373 ± 0.18 log respectively in bacterial counts of young biofilm (till 4^th^ day) (Fig. 3). 5^th^ day onwards a significant reduction of 3.506 ± 0.16 log (*p* < 0.05) was observed in biofilm treated with bacterial depolymerase and gentamicin. In contrast, an insignificant reduction (*p* > 0.05) was observed in biofilm treated with phage depolymerase and gentamicin (Fig. 3).

### Effect of depolymerase and antibiotic alone as well as in combination on disrupted biofilm

**Fig. 4:**
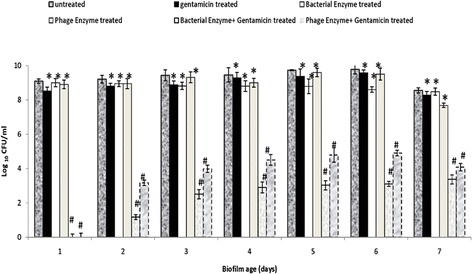
Bacterial count (Log_10_CFU/ml) following treatment of disrupted biofilm of *K. pneumoniae* grown in microtiter plate, with gentamicin (10 μg/ml)/phage/bacterial enzyme (20 units/ml) alone as well as in combination. The experiment was performed thrice in triplicate. Error bars = ± SD; *n* = 3. [*p > 0.05 (gentamicin/bacterial enzyme/phage enzyme treated vs untreated), ^#^ *p* < 0.05 (bacterial enzyme + gentamicin/phage enzyme + gentamicin treated vs untreated)]. Denatured bacterial/phage depolymerase heated at 75 °C for 10 min were used as controls for biofilm treatment. But no effect on CPS content was observed

No significant difference (*p* > 0.05) was observed in average bacterial count of gentamicin treated and untreated disrupted biofilm (Fig. 4). Therefore, disrupted biofilm was treated with phage/bacterial depolymerase alone as well as in combination with gentamicin. In comparison to untreated biofilm, no significant reduction (*p* > 0.05) was observed in the count of disrupted cells (Fig. 4) after treatment with phage or bacterial depolymerase alone. However, on negative staining, bacteria were found to be devoid of capsule compared to untreated cells (data not presented). Treatment with either of the enzyme in combination with gentamicin resulted in complete eradication of 1 day old biofilm (Fig. 4). 2^nd^ day onwards a significant reduction (*p* < 0.05) of 6.679 ± 0.21 log in the average bacterial count was observed after treatment with bacterial depolymerase and gentamicin. In contrast, an average reduction of 5.121 ± 0.18 log was observed in biofilm treated with phage depolymerase and gentamicin in combination (Fig. 4).

### Qualitative analysis of CPS in biofilm

**Fig. 5:**
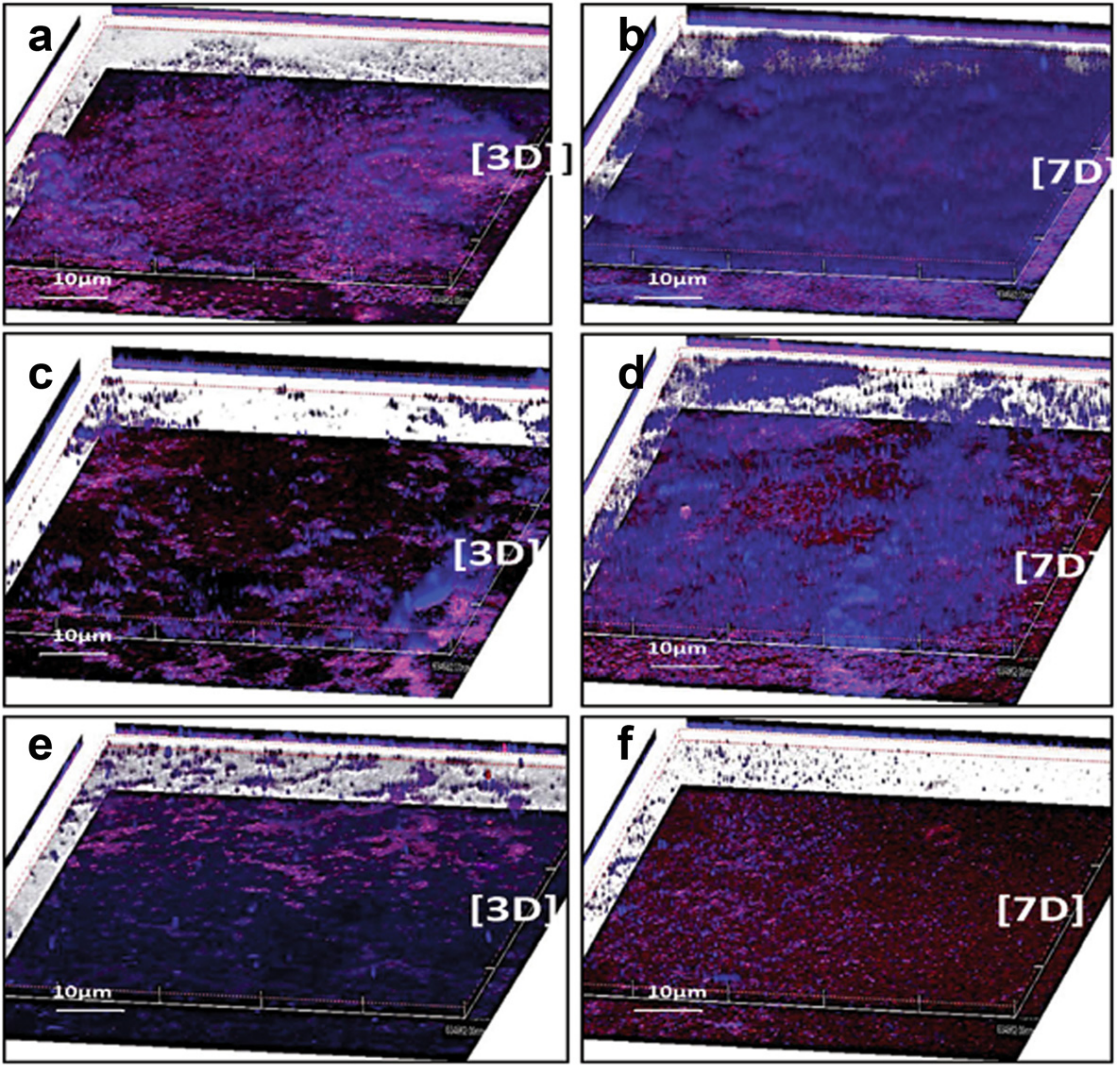
3-D reconstruction of 3 and 7 day old biofilm after staining with calcofluor white and Syto 62 (**a**) 3 day untreated biofilm (**b**) 7 day untreated biofilm (**c**) 3 day biofilm treated with phage depolymerase (**d**) 7 day biofilm treated with phage depolymerase (**e**) 3 day biofilm treated with bacterial depolymerase (**f**) 7 day biofilm treated with bacterial depolymerase. Both the enzymes were used at 20 units/ml. (Magnification 200X). [3D]: 3 day old biofilm, [7D]: 7 day old biofilm, Scale: 10 μm

To determine the ability of bacterial depolymerase in dispersing the polysaccharide matrix enmeshing the bacterial cells, untreated/treated biofilms were visualized under a confocal microscope. Cover slips with biofilm treated with phage depolymerase were also put up simultaneously. In images taken using CLSM, blue regions represent polysaccharide matrix while red regions indicate zones containing cells of *K. pneumoniae* (Fig. 5). Purple regions resulted from overlap of red and blue pixels; representing bacterial cells embedded in polysaccharide. With an increase in biofilm age, an increase in biofilm bacterial mass (red) as well as dense matrix (blue) was observed in untreated biofilm (Fig. 5a and b). This indicated high bacterial density and tightly packed regions of heterogeneously distributed dense polysaccharide in the form of tower or mushroom-shaped clusters in untreated biofilm. A significant change was observed in the enzyme treated biofilm. In comparison to young biofilm treated with phage depolymerase (Fig. 5c), biofilm treated with bacterial depolymerase showed significant decrease in polysaccharide matrix (indicated by blue regions) as well as in surface coverage by bacterial cells (indicated by red regions) (Fig. 5e). In young biofilms, ratio of cell biomass to polysaccharide matrix (integrated red fluorescence density/integrated blue fluorescence density) after bacterial depolymerase treatment was 2.95 U whereas after phage depolymerase treatment it was 1.14 U. In phage depolymerase treated old biofilm, polysaccharide was sparsely distributed in certain areas (Fig. 5d) whereas in bacterial depolymerase treated old biofilm cloud like structure of polysaccharide was rarely present (Fig. 5f). Ratio of cell biomass to polysaccharide in 7 day old biofilm treated with bacterial depolymerase (1.51 U) was significantly higher (*p* < 0.01) than that observed after phage depolymerase treatment (0.58 U) or in untreated biofilm (0.34 U). No significant difference (*p* > 0.05) was observed in the ratio of red to blue fluorescence in phage depolymerase treated (0.58 U) or untreated (0.34 U), 7 day old biofilm.

### Quantification of CPS in treated/untreated biofilms by ELLA

ELLA involving measurement of peroxidase activity after binding of horse radish peroxidase (HRP) labeled lectin (concanavalin A) to sugar residues in polysaccharide was used for quantification of polysaccharide content in biofilm before and after treatment. A significant difference (*p* < 0.05) was observed in the average polysaccharide content (μg/ml) of untreated (1.41 ± 0.26) and bacterial depolymerase treated biofilm (0.45 ± 0.17) (Table 2). In contrast, no significant difference in average polysaccharide content was observed in untreated biofilm (1.41 ± 0.26) and biofilm treated with phage depolymerase (0.90 ± 0.25) (Table 2).

**Table 2:** CPS content (μg/ml) following treatment of *K. pneumoniae* biofilm of different days with phage/bacterial depolymerase (20 units/ml each)

Biofilm age	CPS content (μg/ml)		
Days	Untreated	Phage enzyme treated*	Bacterial enzyme treated^#^
1	0.989 ± 0.15	0.639 ± 0.22	0.279 ± 0.18
3	1.082 ± 0.24	0.846 ± 0.36	0.357 ± 0.17
5	1.589 ± 0.09	0.978 ± 0.24	0.511 ± 0.15
7	2.014 ± 0.35	1.168 ± 0.28	0.656 ± 0.19

Data is presented as mean ± SD (*n* = 3). [*p > 0.05 (average CPS content in phage depolymerase treated vs untreated biofilm), ^#^ *p* < 0.05 (average CPS content in bacterial depolymerase treated vs untreated biofilm)]. Denatured bacterial/phage depolymerase heated at 75 °C for 10 min were used as controls for biofilm treatment. But no effect on CPS content was observed

## Discussion

CPS forms thick bundles of fibrillous structures that cover the entire surface of *Klebsiella* [7]. Because of the inherent tolerance of biofilm to antibiotics, there is a growing need for developing strategies for tackling biofilms [29, 30]. The future of biofilm control lies in using microbes which produce antifouling enzymes [32]. Amylases, proteases, alginate lyases, dispersin, polyglutamic acid depolymerases have been widely accepted for disrupting the polysaccharides of *P. aeruginosa, S. aureus*, *Bacillus* spp. etc. [31]. Various commercially available proteases and polysaccharide degrading enzymes of bacterial and fungal origin, such as glucose oxidase (Novo Nordisk), Lactoperoxidase (Sigma), Pectinex Ultra SP (Novo Nordisk), Mutanase (Novo Nordisk), Dextranase (Novo Nordisk), Subtilisin A (Novo Nordisk) have been used against biofilm formed by *Streptococcus mutans, Staphylococcus* and *Pseudomonas* spp. in various environmental, clinical and industrial settings [32]. In our previous work, *A. punctata* derived capsule depolymerase has been shown to improve gentamicin efficacy during *K. pneumoniae* induced murine infection [19]. Thus, the potential of this depolymerase found to be extensively homologous to protein of unknown function from *A. cavaie* Ae398 [20], in mediating dispersal of biofilm formed by *K. pneumoniae* and improving gentamicin action was evaluated in this study.

Decapsulation of *K. pneumoniae* B5055 after treatment with *A. punctata* derived depolymerase is depicted in Fig. 1. Unlike antibacterial enzymes like endolysins, lysostaphin, lysozyme etc., depolymerase renders the bacterium susceptible to various antimicrobial agents due to digestion of outer CPS [33]. Aminoglycosides have been widely used for inhibiting/restricting the growth of members of enterobactericiae family. Thus, gentamicin was selected for use along with *A. punctata* derived depolymerase. It was used at 10 μg/ml throughout the study, as it is the clinical achievable concentration and optimal concentration approved by CLSI guidelines [34]. Resistance of intact biofilm to gentamicin may be due to the binding of positively charged aminoglycoside to negatively charged biofilm matrix. This has been reported by earlier workers, as tobramycin exhibited a limited or delayed penetration across biofilm of *P. aeruginosa* [35, 36]. Thus, a significant decrease in the penetration of gentamicin with progression in biofilm age observed in this study, was possibly due to binding of gentamicin to the biofilm matrix. Sub-MIC levels of antibiotic thus reaching the biofilm interior may induce matrix synthesis and biofilm formation as reported for *P. aeruginosa* and *Escherichia coli* [29]. Repeated antibiotic dosing also offers no advantage in such cases as it causes saturation of binding sites rather than improving penetration across the biofilm [37, 38]. Thus in this study, young and old biofilm of *K. pneumoniae* were treated with bacterial depolymerase. Although bacterial depolymerase when used alone was not directly bactericidal, a reduction in bacterial count of intact biofilm was observed. This might be due to enzyme catalyzed cleavage of the polysaccharide which caused a removal of the loosely bound, un-encapsulated cells. Earlier studies have reported the use of Dispersin B, ‘a biofilm releasing enzyme’ synergistically with various antimicrobials for the removal of biofilm formed by *S. aureus, S. epidermidis* and *E. coli* [39–41]. Therefore, when bacterial depolymerase was used along with gentamicin, degradation of negatively charged polysaccharide matrix enmeshing bacteria in a biofilm facilitated the transport of positively charged gentamicin across the biofilm. It ensured rapid killing of bacteria present in deeper layers and prevented exposure of bacteria to sub-MIC concentrations of antibiotic as has been reported earlier [42]. Increase in biofilm matrix with an increase in biofilm age which contributed towards inability of gentamicin to penetrate was confirmed by viewing the biofilms by CLSM. The degradation of polysaccharide after treatment with bacterial depolymerase which led to dispersal of loosely bound cells and killing of deep seated bacterial cells by gentamicin was also confirmed through CLSM and ELLA.

Biofilm disruption occurs during its dispersal, due to fluid infusion through them or due to removal of implanted biomaterials like catheters, stents, cardiac pacemakers, endotracheal tubes, joint prostheses, peritoneal dialysis catheters, cerebrospinal fluid shunts etc. Shed bacteria can enter into circulation and cause infections like pneumonia, septicemia and secondary infections especially in immunocompromised individuals [43]. They can also readhere and initiate biofilm formation elsewhere. Stewart and Costerton [44] have reported that antibiotics might kill the free floating bacteria shed from a biofilm. Contrasting results were obtained in our study whereby significant recalcitrance of disrupted biofilm cells to gentamicin was observed. This might be due to the presence of disrupted biofilm cells in clusters. Inability of vancomycin and linezolid to combat disrupted biofilm of methicillin resistant *S. aureus* (MRSA) and methicillin susceptible *S. aureus* (MSSA) at concentrations 1000 times higher than their MICs has also been reported by El-Azizi et al. [27]. Bacterial depolymerase when used in combination with gentamicin caused significant reduction in the count of disrupted cells due to their release from cluster.

Throughout this study, a previously characterized phage borne depolymerase capable of degrading the CPS of *K. pneumoniae* B5055 was used alongwith bacterial depolymerase for biofilm treatment. Even though both the biological entities were used at 20 units ml^−1^, but a better efficacy of bacterial depolymerase over its phage counterpart was visibly clear. During biofilm treatment, phage depolymerase caused ineffective dissolution of polysaccharide especially in older biofilm. Thus, reduction in bacterial count in biofilms treated with phage depolymerase was ~2 log less in comparison to biofilm treated with bacterial depolymerase. Similarly, polysaccharide content after bacterial depolymerase treatment was significantly less in comparison to that observed in biofilm after phage depolymerase treatment. Reason for this anomaly lies in the enzyme kinetics of both the enzymes. Interestingly, a high affinity of the bacterial depolymerase towards its substrate (indicated by its lower K_m_ value = 89.88 μM) over that of phage depolymerase (K_m_ value = 150 μM) and its higher efficiency in liberating reducing sugars [indicated by a higher turnover number (k_cat_ = 285 s^−1^)] over phage depolymerase (k_cat_ =107 s^−1^) was possibly a reason for its higher efficacy than the phage depolymerase (Kinetics of phage depolymerase: Additional file 1: Table S1). Jun et al. [45] have reported a phage endolysin ‘SAL-1’, to possess better efficacy than endolysin ‘LysK’ due to alterations in amino acid residues in catalytic domain. This is the first comparative study on two biological entities capable of degrading the CPS of a gram negative bacterium. The enzyme of bacterial origin was found to show better efficacy over its phage derived counterpart. It is suggested that these biological entities should be further explored for valuable insights into this interesting phenomena.

## Conclusion

In recent times, antimicrobial drug development is increasingly lagging behind the evolution of antibiotic resistance. Considering the degree of virulence possessed by K1 or K2 CPS containing *K. pneumoniae* strains [46] newer antibiofilm therapeutic paradigms are required. The potential of phage borne depolymerase in degrading the biofilm polysaccharide and allowing the phage or other antimicrobials to reach the bacterial cell surface has been widely demonstrated [47–50]. But, obtaining large amounts of phage depolymerases from phage lysates is difficult [33, 51, 52]. Moreover, chances of isolating a natural phage that is specific for a bacteria to be targeted and having the ability to express a relevant polysaccharide degrading enzyme are low [53]. A strategy of engineering bacteria specific phages to express effective polysaccharide degrading enzymes has been looked upon [34]. Use of genetically modified phages which help in overcoming antibiotic resistance [54] or which efficiently kill but simultaneously minimize endotoxin release from the pathogen [55, 56] is also being tried. But, instead of adopting a tedious approach, an enzyme derived from naturally occurring unrelated bacterial genera was used in this study. Such enzymes do not allow generation of resistant mutants, do not involve bacterial lysis and subsequent release of proinflammatory mediators, do not require actively growing cells to exert their action and serve as adjuncts to improve antibiotic action. Promising results obtained with bacterial depolymerase and its better efficacy in comparison to phage depolymerase warrant its further evaluation in *in vivo* studies so that it can be readily incorporated in prophylactic/therapeutic regimes.

## Additional file

Additional file 1:
**Characteristics of bacterial and phage depolymerase** **Figure S1.** Determination of optimum temperature (a) and temperature stability (b) of bacterial depolymerase. Determination of optimum pH (c) and pH stability (d) of bacterial depolymerase. **Figure S2.** Line-weaver Burk plot depicting the kinetics of bacterial depolymerase. K_m_=89.88 μM, V_max_=43.35 μmole/min, K_cat_=285 s^-1^, K_cat_/K_m_= 3.17 s^-1^.μM^-1^. **Figure S3.** Determination of optimum temperature (a) and temperature stability (b) of phage ‘KPO1K2’ derived depolymerase. Determination of optimum pH (c) and pH stability (d) of phage ‘KPO1K2’ derived depolymerase. **Table S1.** Kinetics of phage depolymerase.
