# Supplementary material for: Aeromonas punctata derived depolymerase improves susceptibility of Klebsiella pneumoniae biofilm to gentamicin
Source: BMC Microbiol. 2015 Jun 11;15:119. doi: 10.1186/s12866-015-0455-z (PMC4461996; doi:10.1186/s12866-015-0455-z)
Supplement: Additional file 1: — Characteristics of bacterial and phage depolymerase Figure S1. Determination of optimum temperature (a) and temperature stability (b) of bacterial depolymerase. Determination of optimum pH (c) and pH stability (d) of bacterial depolymerase. Figure S2. Line-weaver Burk plot depicting the kinetics of bacterial depolymerase. Km=89.88 μM, Vmax=43.35 μmole/min, Kcat=285 s-1, Kcat/Km= 3.17 s-1.μM-1. Figure S3. Determination of optimum temperature (a) and temperature stability (b) of phage ‘KPO1K2’ derived depolymerase. Determination of optimum pH (c) and pH stability (d) of phage ‘KPO1K2’ derived depolymerase. Table S1. Kinetics of phage depolymerase. [file 12866_2015_455_MOESM1_ESM.docx]

# Additional file 1

# Clinical, bacteriological, and serological aspects of Klebsiella infections and their spondylarthropathic sequelae.

(d)

(c)

(b)

(a)

**Figure S1: Determination of optimum temperature (a) and temperature stability (b) of bacterial depolymerase. Determination of optimum pH (c) and pH stability (d) of bacterial depolymerase.**

**
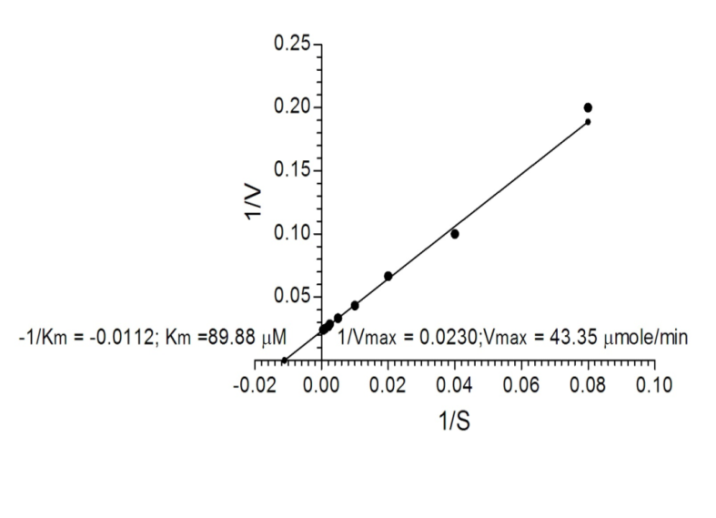
**

**Figure S2: Line-weaver Burk plot depicting the kinetics of bacterial depolymerase. K_m_=89.88 µM, V_max_=43.35 µmole/min, K_cat_=285 s^-1^, K_cat_/K_m_= 3.17** **s^-1^.µM^-1^**

(c)

(d)

(b)

(a)

**Figure S3: Determination of optimum temperature (a) and temperature stability (b) of phage ‘KPO1K2’ derived depolymerase. Determination of optimum pH (c) and pH stability (d) of phage ‘KPO1K2’ derived depolymerase.**

**Table S1: Kinetics of phage depolymerase**

| Characteristic | Phage  Depolymerase |
| --- | --- |
| K_m_ (µM) | 150 |
| V_max_ (µmol min^-1^) | 20 |
| k_cat_ (s^-1^) | 107 |
| k_cat_/ K_m_ (s^-1^.µM^-1^) | 0.71 |
